# Cancer stem cell metabolism: a potential target for cancer therapy

**DOI:** 10.1186/s12943-016-0555-x

**Published:** 2016-11-08

**Authors:** Abhijeet Deshmukh, Kedar Deshpande, Frank Arfuso, Philip Newsholme, Arun Dharmarajan

**Affiliations:** 1 Stem Cell and Cancer Biology Laboratory, School of Biomedical Sciences, Curtin Health Innovation Research Institute, Curtin University, Perth, WA 6102 Australia; 2 School of Biomedical Sciences, Curtin Health Innovation Research Institute, Curtin University, Perth, WA Australia

**Keywords:** Cancer stem cells, Metabolism, Glycolysis, Glutaminolysis, Cancer therapy, Chemo-resistance, Tumour microenvironment, Wnt signalling

## Abstract

Cancer Stem cells (CSCs) are a unipotent cell population present within the tumour cell mass. CSCs are known to be highly chemo-resistant, and in recent years, they have gained intense interest as key tumour initiating cells that may also play an integral role in tumour recurrence following chemotherapy. Cancer cells have the ability to alter their metabolism in order to fulfil bio-energetic and biosynthetic requirements. They are largely dependent on aerobic glycolysis for their energy production and also are associated with increased fatty acid synthesis and increased rates of glutamine utilisation. Emerging evidence has shown that therapeutic resistance to cancer treatment may arise due to dysregulation in glucose metabolism, fatty acid synthesis, and glutaminolysis. To propagate their lethal effects and maintain survival, tumour cells alter their metabolic requirements to ensure optimal nutrient use for their survival, evasion from host immune attack, and proliferation. It is now evident that cancer cells metabolise glutamine to grow rapidly because it provides the metabolic stimulus for required energy and precursors for synthesis of proteins, lipids, and nucleic acids. It can also regulate the activities of some of the signalling pathways that control the proliferation of cancer cells.

This review describes the key metabolic pathways required by CSCs to maintain a survival advantage and highlights how a combined approach of targeting cellular metabolism in conjunction with the use of chemotherapeutic drugs may provide a promising strategy to overcome therapeutic resistance and therefore aid in cancer therapy.

## Background

Chemotherapy, along with radiotherapy and hormone therapy, is the most common treatment for cancer. Due to the side effects of treatment and chemo-resistance of the tumour cells, researchers have shifted their focus to more site-specific treatments in order to achieve better results [1].

**Fig. 1:**
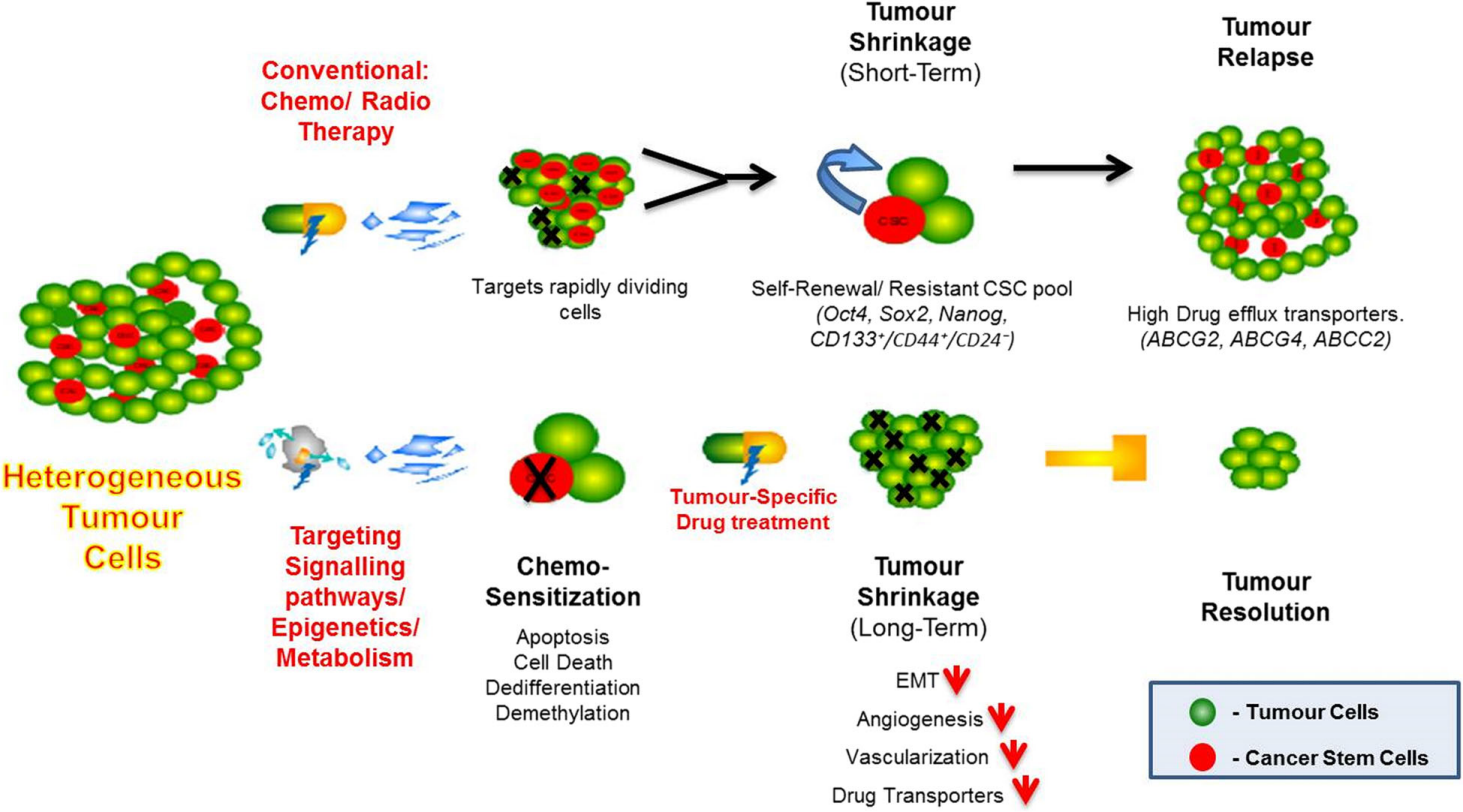
Cancer stem cells (CSCs) are a small sub-population of tumour cells that are highly chemo-resistant and play a prominent role in tumour relapse. The chemo-resistant property of CSCs is believed to contribute to poor prognosis of conventional tumour treatments. Therefore, we rationalise that therapy targeting CSCs would enable chemo-sensitization by affecting downstream cellular signalling pathways of tumour cells and enable the drugs to destroy the tumour bulk.

Over the past decade, a critical role of a small subset of tumour cells, known as cancer stem cells (CSCs), was established in tumour relapse and propagation [2, 3]. Most solid tumours, including breast, brain, prostate, ovary, mesothelioma, and colon cancer contain this small subset of self-renewing tumour initiating cells [4]. Conventional anti-cancer therapies inhibit/kill the bulk of the heterogeneous tumour mass, resulting in tumour shrinkage. However, it has been suggested that later, the CSCs differentiate into tumour cells and are responsible for tumour relapse (Fig. 1). The identification of novel therapies to target CSCs has been the goal of many cancer research laboratories, and recent studies suggest the CSCs undergo metabolic alterations that include low mitochondrial respiration and high glycolytic activity. Exploiting the CSCs' metabolic alterations may provide new effective therapies and diminish the risk of recurrence and metastasis [5, 6].

## Tumour cell metabolism

**Fig. 2:**
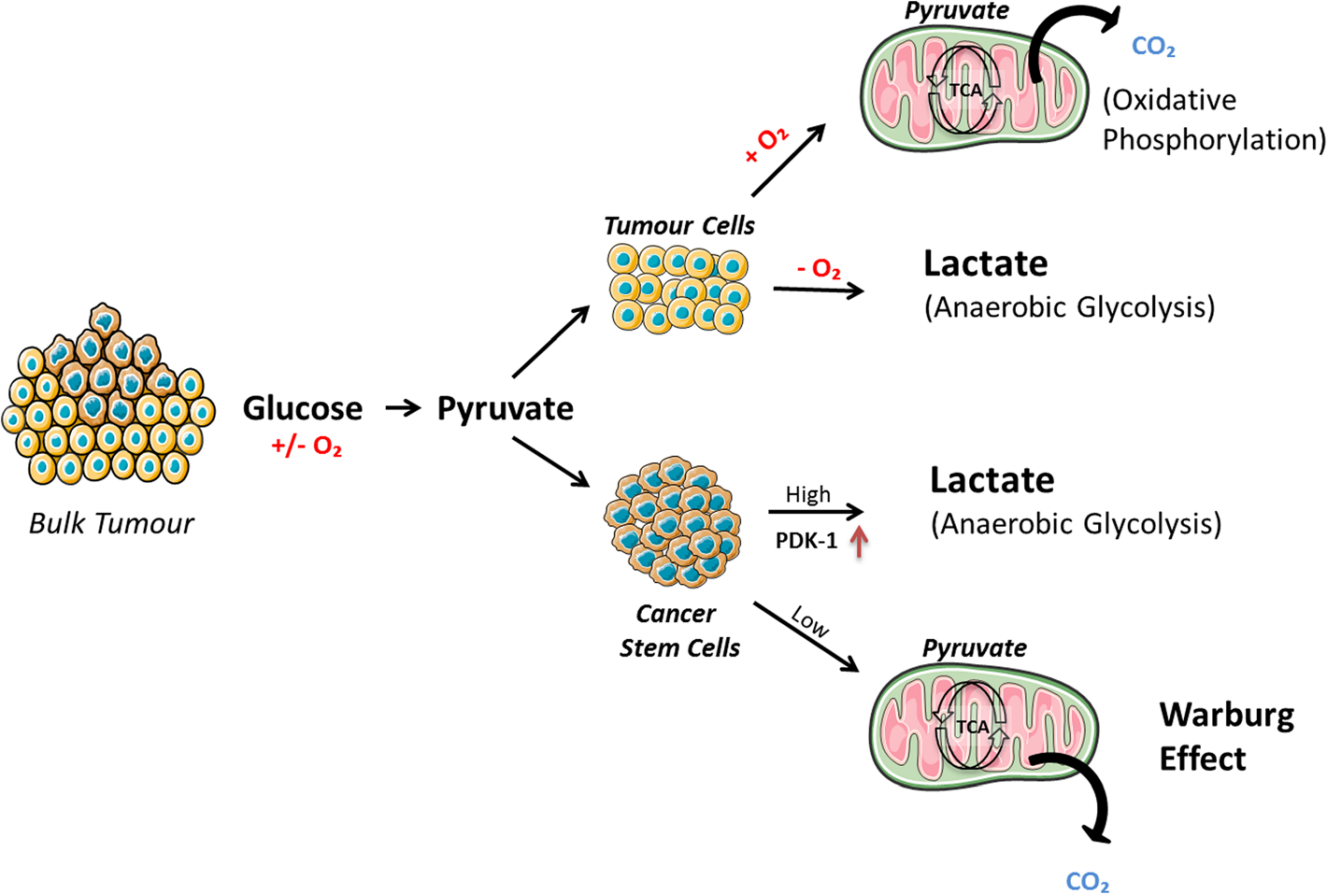
Impact of glucose utilisation by CSCs and non CSCs highlights the difference in their metabolic profiles. Pyruvate enters the TCA cycle to initiate the precursor or supply towards biosynthetic reactions. The Warburg effect in turn activates aerobic glycolysis and lessens mitochondrial respiration, suggesting a preferred choice for proliferation.

To induce their lethal effects and maintain survival, tumour cells alter their metabolism to ensure survival, evade host immune attack, and proliferate [7]. This clever strategy of tumour cells was exposed by Otto Warburg in the 1920s when he proved that, in spite of the presence of abundant oxygen, tumour cells metabolise glucose via glycolysis to produce lactate. They adopt this pathway in order to produce ATP through a fermentation process that is much faster compared to the conventional oxidative phosphorylation (respiration), and also avoids the requirement for mitochondrial oxidative phosphorylation. This meets the requirement for the tricarboxylic acid (TCA) cycle activity to be directed towards biosynthesis rather than ATP production. Inner regions of tumours are known to be hypoxic [7–10]. However, the application of anaerobic glycolysis for energy supply is just one part of the metabolic transformation of tumour cells. In order to multiply and survive, the cell must be able to replicate its genome, protein and lipid content, and other important constituents, and also pass on important biomolecules to daughter cells. To accomplish this, the tumour cells enhance the expression of glucose transporters (GLUTs) and monocarboxylate transporters (lactate/pyruvate) to ensure that glucose is delivered and that lactate is transported out of the cell [7, 11] (Fig. 2). Glutamine (via glutamate) and some of the pyruvate enters the TCA cycle to initiate the precursor supply towards biosynthetic reactions. The theoretical significance of the Warburg effect can be illustrated by the glucose uptake and solvent capacity of the cell cytoplasm, i.e. the maximum number of macromolecules that can be accommodated in the intracellular space. Thus, when the glucose uptake rate is low, glucose uptake capacity is the limiting factor and mitochondrial respiration becomes the preferred source for ATP generation. At a high glucose uptake rate, the cell identifies the solvent capacity as its prime source for generating ATP, which in turn activates aerobic glycolysis and lessens mitochondrial respiration (Fig. 2). Hence, the Warburg effect is the amicable catabolic choice for proliferating tumour cells [12]. The other interesting outcome elicited by the Warburg effect is the creation of a tumour environment that facilitates survival and proliferation of the tumours. In the process of their expansion, the tumours stretch the diffusion limits of their oxygenated blood supply and thus induce hypoxia and stabilize the transcription factor HIF. HIF triggers angiogenesis by regulating various associated factors, especially vascular endothelial growth factor [13, 14]. The other strategy adopted by these tumour cells to maximize their survival and proliferation is to increase their glutamine use for supply of biosynthetic precursors. Glutamine acts as a source of carbon and nitrogen for biosynthetic reactions of cancer cells. It gets converted to glutamate, enters into the TCA cycle, and acts as a precursor for the synthesis of important intermediates such as NADPH, anti-oxidants and amino acids such as α-ketoglutarate, aspartate, glutathione, and nucleic acids. The glutamine is converted to glutamate by the mitochondrial enzyme glutaminase. Glutaminase is highly expressed in rapidly growing tumour cells. Another link between oncogenic activation and tumour cell metabolism was determined when a study established that *c-Myc* increased glutaminase expression by suppressing miR-23a/b [7, 15, 16]. Glutamine may be partially or fully oxidised by tumour cells [17]. It acts as an energy source through catabolism or as a building block via anabolism in the body.

## Cancer stem cells

The origin of CSCs is still unclear and further studies are required in each type of cancer. CSCs are known to remain in G0 phase [18, 19], the resting phase of the cell cycle, and express high drug efflux transport systems. CSCs, being in a dormant state, make it difficult for most anti-cancer drugs that target only proliferative tumour cells. CSCs exhibit specific characteristics such as self-renewal and heterogeneous differentiation capacity, small population (0.001–0.1 %), resistance to chemo/radiotherapy, high metastatic ability, sphere forming ability, and high ABC transporter expression [20, 21]. CSCs are also known to have a high migratory capacity [22], enabling their spread from the primary tumour to secondary sites [23, 24]. Various techniques have been established to isolate CSCs from the tumour mass and characterise them. CSCs are niche forming cells enriched with growth factors, and growing them in serum-free conditions containing growth factors, such as epidermal growth factor (EGF) and basic fibroblast growth factor (bFGF), maintains the undifferentiated stem cell state and induces the proliferation of self-renewing, unipotent CSCs from parental cell lines [4, 25, 26]. CSCs are characterised by specific surface markers such as CD133^+^/CXCR4^+^, CD24^+^/CD44^+^, CD24^+^/CD44^+^/ESA^+^, c-Met^+^/CD44^+^, and ALDH1^+^/CD133^+^ in pancreatic cancer [27, 28]; CD24^−/low^/CD44^+^ in breast cancer; CD44^+^ in colon/ gastric/ head and neck/ovarian cancer; CD34^+^/CD38^−^ in leukaemia cells; CD13/CD45/CD90 in liver cancer; CD117/CD90/EpCAM in lung cancer; CD20/CD166/Nestin in melanoma cancer; and CD133^+^/ABCG2^+^ in Glioblastoma Multiforme [29, 30]. CSCs also express various markers such as CXCR4/ ESA and Nestin [27]. CD44 is one of the most important CSC markers for its role in promoting tumour metastasis and invasion. CD44 has the capability to bind to its primary ligand hyaluronic acid (HA), which initiates CSC attachment to the extracellular matrix and contributes to tumour cell migration [31]. ONCOFID™-S is a conjugate of HA with SN38 (7-ethyl-10-hydroxycamptothecin) and studies have demonstrated that it showed higher anti-proliferative in-vitro activity compared to free SN38 when used against colon, gastric, breast, oesophageal, lung, and ovarian cancer cells, which overexpress CD44 [32, 33]. Therefore, a CD44-targeted therapeutic approach could be utilised for better anti-tumour drug delivery.

The CSCs with CD44^+High^ and CD133^+High^ expression are highly radio-resistant in colon cancer, and they also have higher expression of AKT (AKT1/2) compared to CD44^Low^ and CD133^Low^ cells, indicating their capacity for higher DNA repair and the ability to escape cell death/apoptosis post radiotherapy [34]. Therefore, selective targeting of these markers can be an effective way to deliver cytotoxic drugs to CSCs.

## CSCs and their metabolic alterations

Although much is known regarding metabolic pathways important for tumour survival, the potential for therapeutic metabolic alteration of CSCs still remains under investigation [35, 36]. Recent studies indicate that CSCs have different metabolic properties when compared to the tumour bulk. One such study on brain tumour CSCs revealed that these cells show a low activity of mitochondrial respiration [37]. This finding triggered the need to study the effect of glucose in the microenvironment of CSCs because glucose was estimated to be critical for the CSCs. It was found that CSCs have higher glycolytic rates than other tumour cells [38]. Glucose induced the expression of specific genes in CSCs associated with glucose metabolism and the Akt pathway (*c-Myc*, *GLUT1*, *HK-1*, *HK-2*, and *PDK-1*), which contributes to the rise in the CSC population [38]. Glucose utilisation by CSCs and non CSCs was compared by measuring their glucose consumption and lactate production rates in order to establish evidence for the difference in metabolic profiles of CSCs and the bulk of the tumour. It has been observed that glucose uptake, lactate production, and ATP content are elevated in CSCs as compared to the non CSCs (Fig. 2) [39–41]. Many crucial molecules involved in glucose metabolism have been studied in relation to the metabolism of CSCs, such as hexokinase-1 (HK1), hexokinase-2 (HK2), pyruvate dehydrogenase kinase 1 (PDK1), and glyceraldehyde-3-phosphate dehydrogenase. The PDK1 levels are high in the CSC population (Fig. 2). PDK-1, via the TCA cycle, phosphorylates pyruvate dehydrogenase and suppresses the pyruvate to acetyl-CoA conversion. Furthermore, suppressing the metabolic flow of pyruvate in mitochondria induces the conversion of pyruvate to lactate in the cytosol [38, 42]. HK-1 and HK-2 both catalyse the conversion of glucose to glucose-6-phosphate in glycolysis, but the levels of HK-2 are lower in CSCs while that of HK-1 are higher, suggesting that HK-1 maintains CSCs’ glycolytic activity. Interestingly, *HIF-1α* and *c-Myc* expression (affects HK-2 expression) didn’t change in CSCs and tumour cells. The increase in expression of proteins in the Akt signalling pathway bestows CSCs with a longer life span [8, 38].

Palorini et al. [41] studied the effect of glycolysis inhibition and glucose deprivation on the CSC cell line 3AB-OS, which was derived from the human osteosarcoma cell line MG63. They reported that the 3AB-OS cells require glucose for survival and proliferation. The absence of glucose caused death of the CSC cell line. Glutamine deprivation led to a decline in the MG63 population, which suggested that the 3AB-OS population was not greatly affected by withdrawing glutamine.

Hence, incorporating these features into therapeutic strategies to treat cancer can produce an extensively efficient treatment for various cancers. Also, combining glycolytic inhibition strategies with existing chemotherapy can also help eliminate tumour load completely because the CSCs will also be targeted [41].

## Targeting metabolic regulators

Understanding the mechanism by which CSCs are chemo-resistant and initiate tumour relapse is very important in order to address cancer therapy and to understand CSC biology (Fig. 1). B-cell lymphoma (Bcl-2) protein and its family members are known metabolic regulators, and it is recognised as a crucial mediator of mitochondrial apoptotic signalling. Its metabolic role was confirmed by the presence of Bcl-2 associated death promotor (BAD) in complex with glucokinase [43]. Glucokinase has a low affinity for glucose transporter proteins and is purely substrate driven, making it an ideal substrate sensor to detect glucose in pancreatic Beta cells and hepatocytes [43]. The activation of glucokinase is driven by phosphorylation of BAD by kinases such as Akt. The BAD`s pro-apoptotic capacity is inhibited when bound to glucokinase. However, dephosphorylated BAD, on dissociation with glucokinase, will bind to Bcl-2/xl, initiating apoptosis. Furthermore, it has been shown in some cancers that inhibition of BAD phosphorylation decreases cancer cell survival [44, 45].

The glucokinase complex and BAD accumulation will also promote glycolysis, which favours proliferation and CSC biosynthesis. However, dephosphorylation of BAD shifts the balance towards cell death and inhibits the metabolic signals necessary for high glucose flux to enable cell survival regulation [46].

The Bcl-2 protein family impairs the cell’s ability to release apoptogenic protein cytochrome c from the mitochondria by mediation of the balance between cell survival and apoptosis. It achieves this by binding to the pro-apoptotic proteins Bcl-2 associated X protein (BAX) and Bcl-2 homologous antagonist killer [33, 47]. While the mechanism of Bcl-2 expression in CSC chemo/drug resistance is still unclear, and it might be due to chromosomal translocation or another pathway, it was demonstrated that leukaemia CD34^+^ cells expressed Bcl-2 and Bcl-X [48], and Bcl-2 was highly expressed in breast CD44^+^/CD24^−^/^low^ CSCs [49]. To further understand the role of the Bcl-2 protein family, it was demonstrated that Bcl-2 expression in CD133^+^ human hepatocellular carcinoma cells (HCC) can be regulated by the Akt signalling pathways, the inhibitor specific for AKT1 reduced this cell survival protein expression significantly, indicating that CD133^+^ HCC contribute to chemo-resistance through preferential activation of AKT/PKB and Bcl-2 cell survival response [50].

One of the mechanisms for CSCs to achieve their metabolic shift is through modifications of metabolic and apoptotic roles of Bcl-2 family proteins. The metabolic alterations of this family of proteins may prove potent in increasing cancer cells susceptibility to apoptosis and affecting tumorigenic metabolic reprogramming.

## Targeting drug transporters

CSCs are known to possess a high efflux system that disables the chemo-therapeutic drugs' activity, resulting in the formation of highly drug-resistant tumours [31]. CSCs have been found to express several Adenosine triphosphate–binding cassette (ABC) transporters such as ABCB1/P-gp/MDR1, ABCG2/ BCRP/MXR, and ABCB5 [51, 52]. The ABC transporters are highly dependent on ATP generation in CSCs; thus, targeting CSC metabolism/glycolysis would lead to depleted ATP production and inhibition of ABC transporters. ABCG2 is considered as a high capacity transporter of various substrates including chemotherapeutic drugs [53]. With this in mind, it is has been suggested that ABCG2^+^ tumour cells can represent CSCs, which are known for their drug-resistance. Higher ABCG2 expression has been observed in various CSCs from lung [54], pancreas [55], and liver [56], and is co-expressed with CD133 in melanoma and pancreatic cancer cell lines [57, 58]. It is suggested that ABCG2 expression is upregulated by hypoxia via hypoxia-inducible transcription factor complex HIF-1α and HIF-2α signalling [59].

ABCB1/ P-glycoprotein (P-gp) /MDR1 are known to be expressed in the majority of drug resistant tumours. Being a product of the multidrug resistance (MDR1) gene, it acts as an ATP-dependent efflux pump to various anti-cancer drugs [60]. CSCs derived from pancreatic tumour cells have higher expression of ABCB1 and ABCG2 [61]. Furthermore, the first generation inhibitors (FGI) verapamil and PSC833 were unable to efficiently inhibit mitoxantrone efflux in leukaemic CSCs, showing that high expression of ABCB1 would lead to the development of chemo-resistant cells [62]. Second generation inhibitors (SGI) were structurally modified for more potency, low cell toxicity, and higher specificity, and include dexverapamil [63] and Valspodar (PSC833) [64]. Another SGI, PSC833, showed higher potency compared to the FGI`s, although this is also an inhibitor of cytochrome P-450 and caused drug-drug interaction associated with anti-cancer drugs [65]. Third generation inhibitors (TGI) utilise nano-molar concentrations to have more potency at reversing MDR compared to TGI and SGI. Zosuquidar (LY3359) [66], an oral P-gp inhibitor used in treating acute myeloid leukaemia, significantly increases the uptake of daunorubicin, idarubicin, and mitoxantrone [67]. Another inhibitor, Tariquidar [68], which is used at very low concentrations (25–80 nM), has a high P-gp affinity that inhibits its ATPase activity [69]. Although it has been used as a potent P-gp inhibitor in clinical trials [70], recent studies have shown that Tariquidar is both a substrate and inhibitor of P-gp, depending on its in-vivo dosage [71]. Fourth generation inhibitors (FGI) are natural compounds or plant extracts exhibiting less cytotoxicity and better oral bioavailability. In-vitro analysis showed MDR reversal of ABC drug transporters when treated with extracts of Chinese herbal plants such as flavonoids or stilbenes [72]. Some natural compounds such as trabectedin, cytarabin, and halaven, have been approved for clinical use based on their strong MDR reversal activity by impacting on ABC drug transporters [73–76].

ABCB5β (a half-transporter) has been found in malignant melanoma and breast cancer, and is known to mediate doxorubicin resistance [77]. The ABCG5^+^ cells represent 2–20 % of the melanoma tumours and have been shown to successfully recapitulate the tumour in immuno-deficient mice; however, these tumours were unable to regenerate ABCG5^+^ cells, suggesting their limited stemness capacity [20].

Inhibition of ABC transporters can also cause toxicity to a patient’s normal stem cells, since these have an enhanced DNA repair mechanism, particularly bone marrow-derived stem cells. In addition, ABCG2 and ABCB1 play a pivotal role in maintaining the blood brain barrier, and interfering with their normal function could have drastic consequences [78].

## Targeting the tumour microenvironment

Tumour progression is due to adaptive cellular responses such as dormancy, invasiveness, and chemo-resistance in the tumour metabolic microenvironment [79]. Adaptive behaviour of CSCs in this heterogeneous microenvironment is one of the characteristics of CSCs [80]. The tumour microenvironment plays a pivotal role in cancer cell progression, particularly for CSCs, and it mostly involves hypoxia, nutrition, and low pH [81].

Hypoxia in the tumour microenvironment allows pro-angiogenic factors to stimulate new vessel growth within the solid tumour, although the vessels tend to be immature and exhibit poor perfusion [82]. Hypoxia, due to its spatial and temporal heterogeneity in tumours, is difficult to treat [83]. The hypoxic response within the microenvironment is regulated by Hypoxia inducible transcription factors, HIF-1α/ HIF-2α. The migration, glycolytic, angiogenic, and cell survival pathways constitute the transcription targets of HIF1α [84]. Hence, targeting HIF1α is a potential therapy for cancer treatment.

In hypoxic stress, the endoplasmic reticulum (ER) is inhibited, activating the Unfolder protein response (UPR). The UPR maintains ER homeostasis and its disruption initiates apoptosis. Aberrant activation by the UPR in the absence of the two ER membrane proteins PERK (PKR-like ER kinase) and IRE-1 (inositol-requiring) results in increased hypoxia and reduced growth rates [85, 86]. The UPR is an important cellular response mechanism in cancer, playing a role in calcium homeostasis, redox status, and glucose deprivation within the tumour.

Another potential target within the microenvironment is mammalian target of rapamycin (mTOR). During cell stress, nutrient and energy depletion within the solid tumour, mTOR activates the signalling cascades responsible for metabolism and cell survival mechanisms [87, 88]. The anti-diabetic drug metformin has exhibited potential anti-tumour activity; it reduces blood glucose levels, thereby inhibiting gluconeogenesis, and initiates AMPK (AMP-activated protein kinase) activation [89]. AMPK regulates the mTOR activity through activation of the tuberous sclerosis protein 1 complex (TSC1/2) [90].

The microenvironment of tumours is more acidic (pH 6.5–6.9) compared to normal tissues (pH 7.2–7.5), resulting in tumours having poor vascular perfusion and increased glycolytic flux [91, 92]. Knowing that tumour invasiveness is more active in an acidic microenvironment [93, 94], manipulating the tumour microenvironment pH by orally distributed systemic buffers is an effective way to increase the extracellular pH of tumours [95, 96].

## Targeting glycolytic enzymes to reduce chemo-resistance in CSCs

Most cells satisfy their energy demands through glucose catabolism, which is subject to complex regulation. To inhibit glucose catabolism through the central pathway of glycolysis, various glycolytic enzymes or transporters must be targeted such as GLUT 1 - 4 [10], hexokinase [97], pyruvate kinase M2, and lactate dehydrogenase A [98].

Cancer cells have the ability to alter their metabolism in order to fulfil bioenergetic and biosynthetic requirements. The extracellular environment can be acidified by what is known as the ‘Warburg effect’ (a term referring to high levels of glycolytic pathway flux, even under aerobic conditions). When HIF-1α induces the expression of carbonic anhydrases, and there is an interaction with extracellular acidification, the pH ratio between the intracellular and extracellular environment is altered [99–102].The resultant pH shift affects drug absorption within the cell. At the same time, glycolytic adenosine triphosphate (ATP) production and the transporter induced over-expression of HIF-1α contribute to a decrease in the cytoplasmic retention of anti-cancer agents [37, 103].

## Targeting mitochondrial respiration

The distinct metabolic profile of CSCs has been reported in a few types of cancer, demonstrating CSCs to be more dependent on mitochondrial respiration and less on glycolysis [104]. CSCs prefer oxidative phosphorylation (OXPHOS) for energy production in lung cancer [105], glioblastoma [106], pancreatic ductal adenocarcinoma (PDAC) [104, 107], and leukemic stem cells [108]. The finding in PDAC cells and PDAC-CSCs demonstrates that unlike other highly glycolytic tumour cells, the PDAC-CSCs do not depend on lactate production to generate NAD+ for anabolic respiration to support continued glycolysis and are more dependent on mitochondrial respiration [104]. OXPHOS inhibition impacts directly to the CSCs’ sphere formation capacity and tumorigenic potential, indicating extreme sensitivity to mitochondrial function inhibition [104, 107]. The CSCs’ strong dependence on mitochondrial electron transport chain activity on autophagic and catabolic processes makes them more resistant towards nutrient and environmental factors [104, 107]. In normal and leukaemic stem cells, a dependence on OXPHOS for energy production demonstrates the importance of mitochondrial respiration [108–111]. These findings imply an alternative approach to target tumour relapse by targeting OXPHOS in association with oncogenic pathway inhibitors in pancreatic cancer [104].

## Glutaminolysis in cancer metabolism

Cancer cells metabolise glutamine, as well as glucose, to grow rapidly because it provides the required ATP and essential biomolecules such as proteins, lipids, and nucleic acids [112]. Glutamine influences the signalling pathways required for cancer cell proliferation, survival, and metabolism through regulation of mitochondrial reactive oxygen species (ROS) production [113, 114]. Activation of the PI3-Kinase-Akt pathway results in increased production of ROS in mitochondria through metabolic pathways [115]. Glutamine is first converted to glutamate by the enzyme glutaminase, and then glutamate is converted to α-ketoglutarate (αKG) by the action of glutamate dehydrogenase (or an aminotransferase). The rapidly growing tumour cells use glutamine as a carbon source for energy production and for the replenishment of TCA cycle intermediates such as pyruvate, oxaloacetate, and αKG to make up for the constant loss of citrate, which is exported out of the mitochondria for lipid synthesis. It has been observed that glutamine withdrawal in cells with increased *c-Myc* expression led to the death of the oncogenic cells [16]. Thus, it can be confirmed that cancer cells employ glutamine to provide substrates for the TCA cycle [113, 116]. Further studies have also demonstrated that the oncogene *c-Myc* impacts glutamine metabolism, thus stimulating the glutamine transporters SLC5A1 and SLC7A1 and, as a result, promoting the expression of glutaminase 1 by suppressing the expression of miR-23A and miR-23B [39].

These data provide a concrete platform to include glutamine metabolism in cancer as an integral part of cancer therapeutic strategies. Glutamine analogues such as 6-diazo-5-oxo-L-norleucine (L-DON), acivicin, and azaserine were found to demonstrate anti-cancer activities but were not formulated into drugs due to their neuro- and gastrotoxicity [117]. However, it has been shown that inhibition of glutamine metabolism via L-DON was able to reduce cancer metastasis in a VM-M3 mouse model [39, 118]. Zhou et al. [119] performed a proteomic analysis in pancreatic ductal adenocarcinoma that revealed the role of glutamine metabolism in cancer. They found that the level of glutaminase in the cancer cells was much higher compared to the normal ductal cells. In addition, the concentration of other enzymes such as cytidine triphosphate synthase, guanine monophosphate synthetase, and asparagine synthetase, which use glutamine as substrates, was found to be elevated in pancreatic cancer. This indicates that the high utilisation rates of glutamine by cancer cells are required to satisfy their need of nitrogen and energy for uninterrupted, fast growth. Paediatric acute leukaemia has been successfully treated by L-asparaginase, which catalyses the hydrolysis of asparagine to aspartic acid. This enzyme is also capable of hydrolysing glutamine to glutamic acid and ammonia, thus reducing blood glutamine levels [39].

Histone deacetylase (HDAC) inhibitors such as phenylbutyrate have been used pharmacologically to inhibit the invasive properties of breast and prostate cancer by inducing apoptosis and depleting the blood glutamine levels [120, 121]. It is generally used to treat hyperammonemia in urea cycle disorders, but it also brings down the level of glutamine in the plasma by forming a conjugate and thus helps curb tumour growth [122]. The glutamine transporters SLC1A5 (ASCT2) and SLC1A7, which are over-expressed in various human cancers such as colon, liver, colorectal adenocarcinomas, glioblastoma multiforme, and melanoma, have been attractive targets due to their role in cell survival signalling and also being a major source of glutamine delivery [123]. IL-γ-glutamyl-p-nitroanilide has been shown to inhibit SLC1A5 (ASCT2) and cause autophagy in cancer cells [39, 116]. A chemical compound termed 968 exhibited anti-glutaminase activity, which in turn suppressed the oncogenic transformation by *c-Myc* via down regulation of miR-23, which has been seen in prostate cancer and human B-cell lymphoma [39]. Another compound, Bis-2-(5-phenylacetamido-1, 2, 4-thiadiazol-2-yl) ethyl sulphide, also exhibited inhibitory effects on glutaminase, thus repressing glutamine availability to the cancer cells [117]. Ongoing and future work would aim at presenting a more detailed picture of glutamine metabolism and its involvement in cancer, which would help develop safe and effective glutamine inhibitors.

## Conclusion

The targeting of CSCs is emerging as a novel therapy to eradicate the progression of various cancers. The inefficiency of traditional anti-cancer therapies lay the stepping stone for studying the metabolism of cancer cells and the pathways controlling and regulating their growth and proliferation, and converting them into formidable treatment options. Targeting the special metabolic traits of CSCs would enable the basis for the development of new therapeutic strategies to inhibit the bulk of the tumour. Clinically, targeting the CSCs resistant towards therapy and metastasis would enable long term disease free survival for the patients.

Though, drug development for CSC metabolism is gaining wide interest, it is still controversial issue as there are studies contradicting the glycolytic phenotype of CSC and oxidative state of CSCs. On the other hand, cancer cell metabolism has emerged to be one of the most fascinating and promising areas in cancer therapy research. The current research focuses on trying to understand the metabolic demands and profile of cancer cells, and design drugs accordingly in order to add a new exciting chapter to cancer treatment. Also, drugs targeting cancer metabolism can be employed for multiple cancers, which can possess a broad spectrum of activity, and are indeed under clinical trials that will likely result in new treatment options in the future [124]. Despite the limited research on the role of metabolism in CSCs and their ability to self-renew, tumour initiation, differentiation capacity, chemo-resistance and survive therapy, targeting CSCs metabolism holds great promise in translating cancer treatments. Though, combinatorial treatments involving both standard chemotherapeutic drugs and chemo-sensitizing agents on CSCs would probably be the most efficient CSC-targeted therapy (Fig. 1).

## Abbreviations

ABC: ATP binding cassette
Bcl-2: B-cell lymphoma
CSCs: Cancer stem cells
HK: Hexokinase
OXPHOS: Oxidative phosphorylation
PDAC: Pancreatic ductal adenocarcinoma
PDK1: Pyruvate dehydrogenase kinase 1
PTCH1: Patched homologue
SDF1: Stromal cell derived factor 1
TCA: Tricarboxylic acid cycle
αKG: α-ketoglutarate
